# Analysis of the risk factors for secondary hemorrhage after abdominal surgery

**DOI:** 10.3389/fsurg.2023.1091162

**Published:** 2023-06-06

**Authors:** Tianshu Pang, Zhengrong Wu, Hongfen Zeng, Xiangyu Zhang, Mengya Hu, Liping Cao

**Affiliations:** ^1^ Department of General Surgery, School of Medicine, Sir Run Run Shaw Hospital, Zhejiang University, Hangzhou, China; ^2^ Department of Nursing, School of Medicine, Sir Run Run Shaw Hospital, Zhejiang University, Hangzhou, China

**Keywords:** secondary hemorrhage, abdominal surgery, bleeding, hemostasis, predictors

## Abstract

**Introduction:**

This study aimed to conduct a clinical review and analysis to recommend options for the prevention and treatment of postoperative hemorrhage.

**Patients and Methods:**

A total of 138 patients who experienced postoperative hemorrhage after abdominal surgery in the period between January 2015 and December 2020 at the Sir Run Run Shaw Hospital, affiliated to Zhejiang University School of Medicine, participated in this study. They were divided into a group with primary bleeding only and a secondary bleeding group. Univariate and multivariate statistical analyses were performed, followed by plotting of cumulative hazard and survival curves for the two groups.

**Results:**

The main factors of interest found to be associated with secondary hemorrhage were duration of the operation, the time of the first bleeding incident, intervention time, performance of combined organ resection, use of surgical intervention, occurrence of abdominal infection, admission to the intensive care unit (ICU), postoperative length of stay, and total hospitalization expenses. Among these, a long operative duration (>5 h) and an extended intervention time (>5 h) were identified as independent predictors of risk of secondary hemorrhage.

**Conclusions:**

Secondary hemorrhage after abdominal surgery is mainly associated with subjective human factors, and it is an important cause of poor prognosis and even death. Proper reductions in operation time and implementation of a quick response to bleeding are the key factors in tackling bleeding. Further reduction in the rates of postoperative hemorrhage and mortality will require a concerted effort by surgeons in terms of both intraoperative surgical techniques and postoperative management.

## Introduction

Abdominal surgery is one of the most common procedures in general surgery. Intra-abdominal hemorrhage after abdominal surgery refers to cases in which there is postoperative drainage of hemorrhagic fluid in the abdominal drainage tube or imaging evidence indicating intra-abdominal hemorrhage with abdominal distension and a decrease in hemoglobin, which can also be confirmed by reoperative exploration, imaging examination, or endoscopy (1). If not timely detected, postoperative hemorrhage will lead to increased complications and severe consequences even if the surgery performed was minimally invasive. This makes postoperative hemorrhage one of the leading causes of death after abdominal surgery (2). Furthermore, heavy secondary postoperative bleeding is often ignored, which can result in a high incidence of complications as well as a prolonged hospital stay. When compared with primary bleeding, secondary bleeding is more likely to cause hemorrhagic shock, disseminated intravascular coagulation, and multiple organ failure, eventually leading to increased mortality.

In this study, patients with postoperative abdominal hemorrhage admitted to a large public hospital were selected as research participants. The clinical history of the patients was determined and a clinical analysis was conducted to identify options that can be recommended for the prevention and treatment of postoperative hemorrhage.

## Patients and methods

### General information

**Figure 1:**
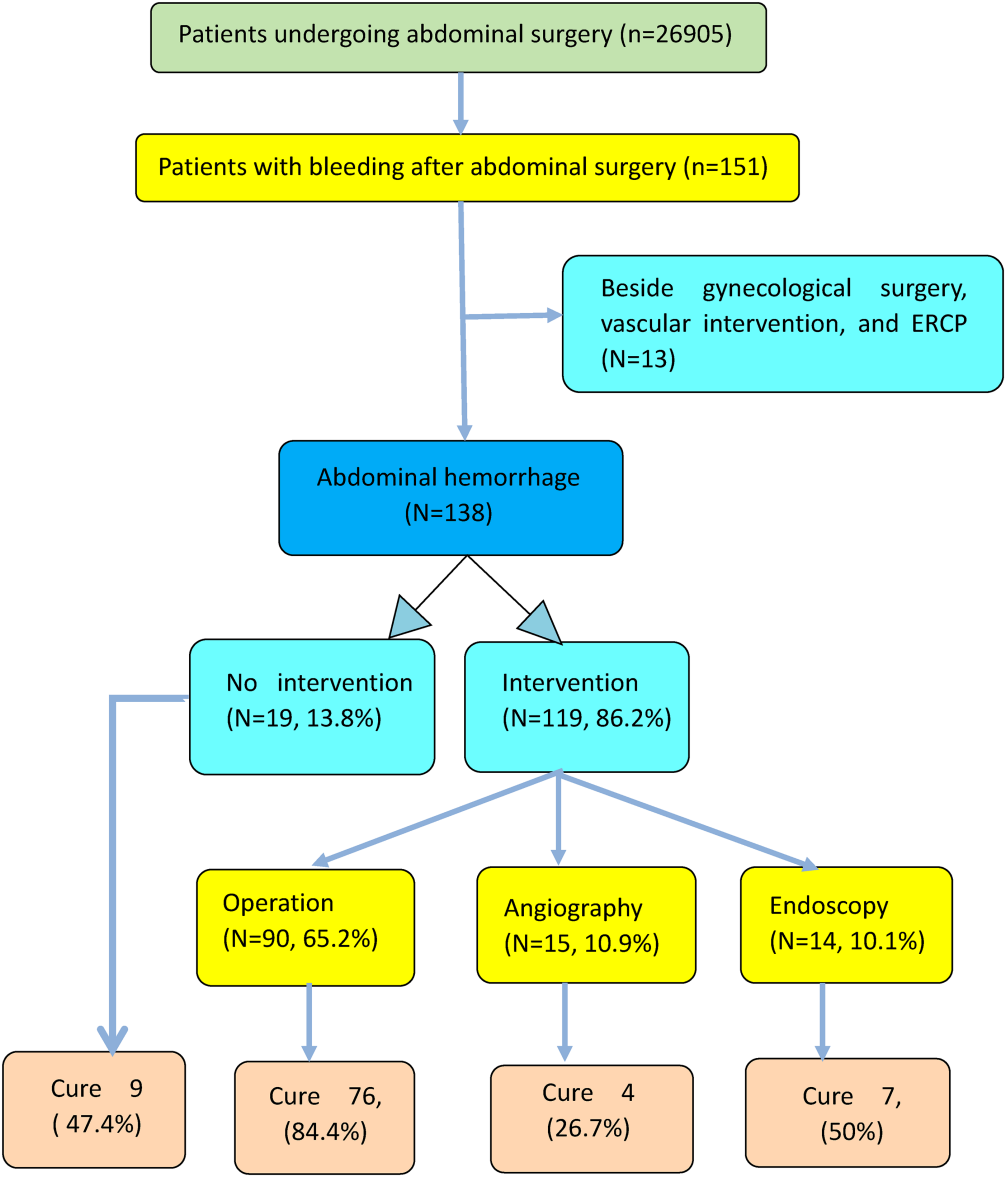
Screening procedure of patients and hemostasis measures.

A retrospective study was conducted; 138 patients with postoperative bleeding were selected as participants, after several layers of screening, from a total of 26,905 patients who had undergone abdominal surgery (Figure 1). The records of these 138 patients who experienced abdominal hemorrhage in the period between January 2015 and December 2020 were collected from the clinical database at the Department of General Surgery, Sir Run Run Shaw Hospital, School of Medicine, Zhejiang University. The criteria for inclusion in this study were as follows. (1) Abdominal surgery included surgery of the abdominal cavity and pelvis, excluding the parts above the diaphragm. (2) All the patients included were followed up from starting hospitalization to discharge from the hospital or died in the hospital, and those that occurred bleeding once more within 2 weeks after discharge were excluded. (3) Patients who underwent endoscopic submucosal dissection, endoscopic retrograde cholangiopancreatography, and biliary stent implantation were not excluded from this study.

Intra-abdominal hemorrhage can be diagnosed based on any two or three of the following five conditions: (1) postoperative shock, apathy, clammy skin on the extremities, and systolic blood pressure <90 mmHg or pulse pressure <20 mmHg and heart rate >100 beats/min, or shock index (shock index = pulse rate/systolic blood pressure) ≥1; (2) discharge of a large amount of blood (≥50 ml/h) from the abdominal drainage tube; (3) non-clotted blood after abdominal puncture; (4) progressive decrease in hemoglobin levels; (5) CT imaging and B-ultrasound indicating a large effusion or a progressively increased effusion.

### Research methods

Patients were divided into a primary bleeding group (those with successful hemostasis and in whom bleeding occurred only once) and a secondary bleeding group (those who could not be classified under the first category, and in whom bleeding occurred at least twice). General data and perioperative information on these two groups were collected and compared; this comparison indicated that the major observation indicators were postoperative hemostatic effect and postoperative outcome.

During surgical hemostasis, active bleeding was observed under laparoscopy or laparotomy, and hemostasis was considered successful once there was no bleeding in the operation area. This assessment was made regardless of the use of ligation and suture or electrocoagulation and cauterization. During interventional hemostasis, the relevant bleeding vessel was identified and confirmed by angiography. This was used to verify that the overflow contrast agent disappeared after embolization, thereby confirming the success of this process. During endoscopic treatment, vascular bleeding was identified in the lumen of the digestive tract, and hemostasis was considered successful if no bleeding was observed after clipping with titanium clips or snare ligation.

The research protocol was reviewed and approved by the Research Ethics Committee of Sir Run Run Shaw Hospital, School of Medicine, Zhejiang University. The Institutional Review Board of the hospital approved the request to access the hospital database to identify inpatients with abdominal bleeding. The local ethics committee of the hospital also approved the study protocol. The protocol in this retrospective clinical study was followed in accordance with the principles of the Helsinki Declaration.

### Statistical analysis

The risk predictors for postoperative bleeding were determined via univariate and multivariate analyses using logistic regression methods. Continuous variables are reported in the form $(\bar{X}\pm S)$ and compared using an independent samples *t*-test; categorical variables are reported in the form of frequencies and compared using Pearson's chi-squared test. The significant predictors with potential relevance were determined using multivariate logistic regression analysis. The related variables were subjected to backward conditional stepwise regression using unconditional binary logistic regression analysis in order to estimate the relevant odds ratio (OR) and corresponding 95% confidence interval (95% CI) for quantification of the risk associated with postoperative secondary hemorrhage. The Kaplan–Meier survival analysis function was applied to plot trends in the cumulative hazard as well as the survival curves of the two groups. All analyses were carried out using PASW 20.0 statistical analysis software (SPSS, Chicago, IL, USA). *P* values were based on two-sided tests, with *P* < 0.05 regarded as statistically significant, and *P* < 0.001 as indicating significant differences.

## Results

### General information

A total of 138 eligible patients with postoperative bleeding after abdominal surgery, consisting of 99 men and 39 women with ages ranging from 24 to 89, were included in this study. As shown in Table 1, the mean age of the primary bleeding group was 61.55 ± 13.8 years, whereas that of the secondary bleeding group was 63.91 ± 10.6 years. No statistical difference in gender and age was observed between the two groups (*P* > 0.05, indicating that the data were comparable. The surgical history of the patients was as follows: 38 cases of pancreaticoduodenectomy, 8 cases of pancreatic body tail resection plus splenectomy, 22 cases of hepatectomy, 29 cases of gastrectomy, 14 cases of colorectal resection, 19 cases of cholecystectomy plus choledocholithotomy, and 8 cases of abdominal tumor resection and intestinal anastomosis.

**Table 1:** Analysis of clinical data in cases of postoperative bleeding after abdominal surgery.

Category	Primary bleeding only (*N* = 106)	Multiple bleeding events (*N* = 32)	*χ*^2^/t	*P*
Sex			0.184	0.668^a^
Male	77	22		
Female	29	10		
Age (years)	61.55 ± 13.81	63.91 ± 10.55	0.811	0.368
≤65	50	18		
>65	56	14		
History of abdominal surgery	34	7	1.225	0.268^a^
Hypertension	38	12	0.029	0.865^a^
Operative method			0.003	0.956^a^
Laparoscopic surgery	69	21		
Exploratory laparotomy	37	11		
Preoperative prothrombin time (s)	13.46 ± 1.43	13.28 ± 0.90	0.687	0.493^b^
Coagulation function			0.033	0.857^a^
Normal	88	27		
Abnormal	18	5		
Tumor size (cm)	3.93 ± 2.77	4.19 ± 3.15	−0.390	0.697^b^
Number of lymph nodes	19.06 ± 14.67	17.43 ± 10.01	0.491	0.625^b^
TNM staging				0.451^c^
Benign	42	9		
Stage I	40	12		
Stage II	10	6		
Stage III	9	5		
Stage IV	5	0		
Combined organ resection	45	20	3.965	0.046^a^
Operative duration (h)			6.805	0.009^a^
≤5	61	10		
>5	45	22		
Intraoperative blood loss (ml)	312.9 ± 502.1	381.1 ± 445.2	−0.691	0.491^b^
Intraoperative blood transfusion	28	11	0.768	0.381^a^
Time of first bleeding incident (d)	3.6 ± 4.8	7.5 ± 7.7	−2.759	0.009^b^
Intervention time (h)			9.486	0.002^a^
≤5	92	20		
>5	14	12		
Hemostatic approach			8.464	0.004^a^
Surgical intervention	76	14		
Non-surgical treatment	30	18		
Abdominal infection	21	12	4.227	0.040^a^
Pulmonary infection	8	6	3.384	0.066^a^
Admission to intensive care unit		17.312	<0.001^a^	
Yes	45	27		
No	61	5		
Died			10.563	0.001^a^
Yes	11	11		
No	95	21		
Length of postoperative stay (d)	25.0 ± 20.8	48.4 ± 29.5	−4.176	<0.001^b^
Total hospitalization expenses (ten thousand yuan)	15.64 ± 10.32	37.3 ± 23.73	−5.020	<0.001^b^

^a^ *χ*^2^ test applied.

^b^ t-test applied.

^c^ Unconditional binary logistic regression.

Time of first bleeding incident refers to the interval between the end of abdominal surgery and the first detection of bleeding. Intervention time refers to the time between the discovery of bleeding and clinical intervention.

Of all patients included in this study, 106 (76.8%) had primary bleeding only, and 32 (23.2%) had secondary bleeding. In 116 cases (84.1%), the patient was ultimately cured, while 22 patients (15.9%) who received at least one intervention were not cured and eventually died. Secondary surgery was performed in 90 patients, with successful hemostasis observed in 76 (84.4%); interventional embolism was performed in 15 patients, with successful hemostasis achieved in 4 (26.7%); endoscopy was performed in 14 patients, with successful hemostasis achieved in 7 (50%); and the remaining 19 patients received conservative treatment, with 9 being completely cured (47.4%) (Figure 1).

### Univariate and multivariate analyses for postoperative hemorrhage

**Figure 2:**
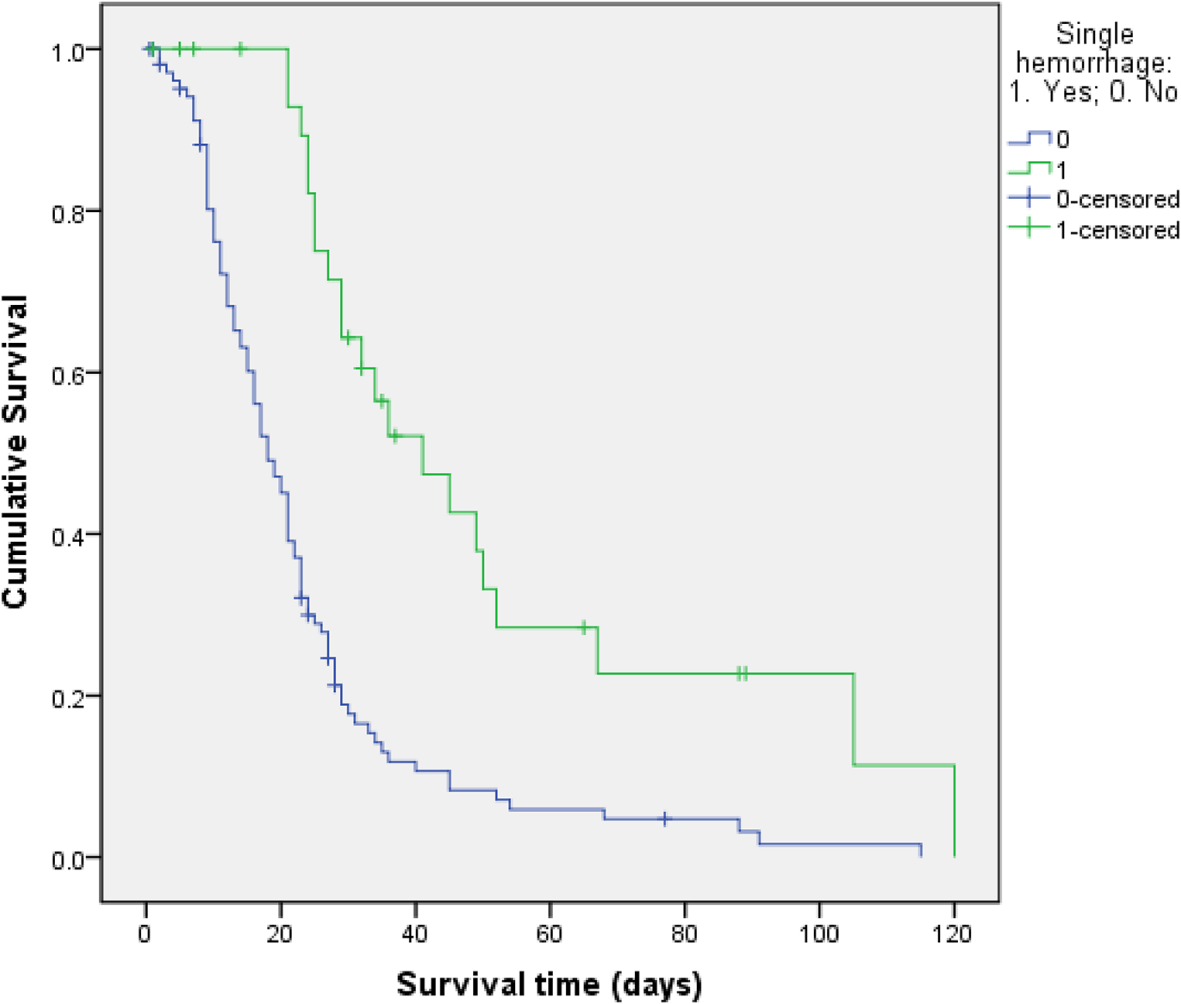
Survival function for patterns.

**Figure 3:**
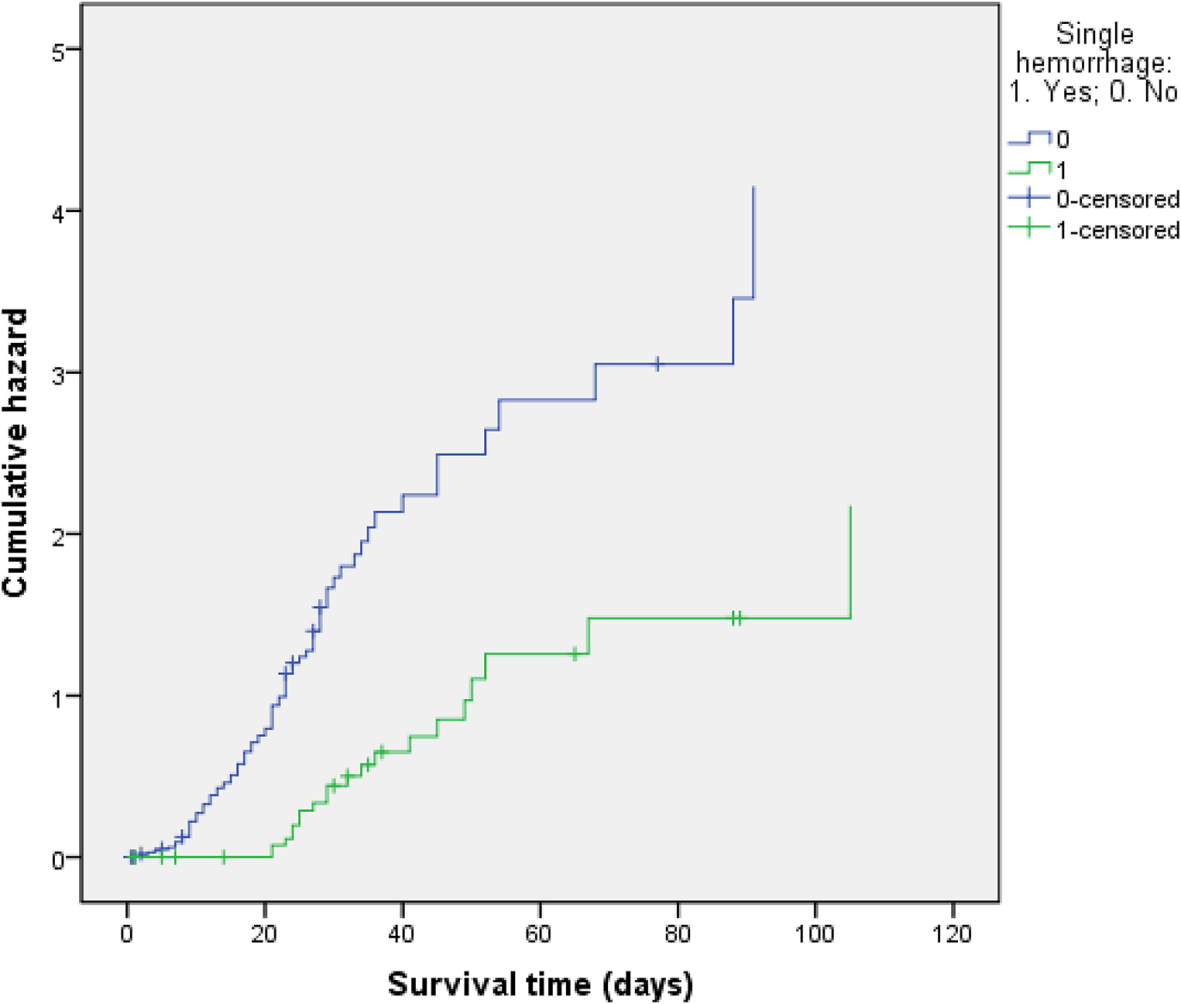
Hazard function for patterns.

As shown in Table 1, the univariate analysis indicated that the items on which the two groups were statistically different were the following: whether combined organ resection was undertaken; duration of operation, the time from the end of the operation to the first bleeding event; intervention time; hemostatic approach; abdominal infection; intensive care unit (ICU) admission rate; operative mortality; postoperative length of stay; and total hospitalization expenses (*P* ≤ 0.05). Specifically, secondary hemorrhage led to a significant increase in the ICU admission rate, operative mortality, postoperative length of stay, and total hospitalization expenses. Therefore, six factors were selected as major predictive indicators of postoperative secondary hemorrhage, namely, undertaking of combined organ resection, longer operation time (>5 h), delayed bleeding (late detection of first bleeding event, i.e., >24 h), intervention time >5 h, use of a non-surgical approach, and occurrence of abdominal infection. Other factors, such as the presence of a history of abdominal surgery, hypertension, use of laparoscopic surgery or laparotomy, preoperative coagulation function, tumor size, number of lymph nodes, TNM stage of tumor, occurrence of pulmonary infection, intraoperative blood loss, and administration of blood transfusion, did not differ significantly between the two groups (*P* > 0.05). We also compared all ten surgeons who performed the 138 operations and found that which surgeon performed the operation had little to do with whether secondary bleeding occurred (*P* > 0.05). Notably, the survival function curve was significantly lower in the primary hemorrhage group, while the risk of death was significantly higher in the secondary hemorrhage group (Figures 2, 3).

In the multivariate logistic regression analysis, the statistically significant predictive indicators (as identified in the screening analysis above) were subjected to backward stepwise regression, with the addition of gender and age to avoid confounding bias. As shown in Table 2, after the removal of mixed factors, two risk factors, operative duration >5 h and intervention time >5 h, were independently associated with secondary bleeding after abdominal surgery; the ORs (95% CI) associated with these two independent significant predictors were 2.776 (1.147–6.721) and 4.231 (1.615–11.084), respectively.

**Table 2:** Multivariate logistic regression analysis comparing the two groups.

Risk factors	*P*	OR	95% CI
Operative duration >5 h	0.024	2.776	1.147–6.721
Intervention time >5 h	0.003	4.231	1.615–11.084
Abdominal infection	0.070	2.355	0.934–5.941

95% CI=95% confidence interval.

## Discussion

Postoperative abdominal hemorrhage is a common complication, with a majority of patients requiring surgical, endoscopic, or embolic intervention. Some of the patients with this condition even require more than one procedure. Similarly to the secondary bleeding observed following hypertensive intracerebral hemorrhage, as proposed by Brott (3), secondary bleeding may also occur after abdominal surgery. Such bleeding occurs in other parts of the abdominal cavity after stabilization of the first bleeding incident requiring hemostasis measures, and is known as secondary bleeding. In this study, the location of the secondary bleeding differed from that of the primary bleeding incident in all 32 patients in whom multiple bleeding events occurred. If the primary bleeding is not stopped, the severe consequences of rebleeding could lead to life-threatening outcomes, thereby significantly increasing the mortality rate. In this study, among the 22 patients who died, 13 died of hemorrhagic shock and 6 died of hemorrhagic shock combined with infectious shock, accounting for 86.4% of the total deaths. This shows that severe postoperative hemorrhage has a major negative impact on prognosis. Unsuccessful hemostasis can lead to severe consequences, such as disseminated intravascular coagulation, multiple organ failure, and even death.

In this study, the significant predictors of secondary hemorrhage were duration of operation >5 h and intervention time >5 h; these were independent risk factors. The effect of longer operation time can be attributed not only to the difficulty of the operation but also to hesitation on the part of the surgeon or excessive dissection of vessels. Difficult and complicated operations generally require more blood vessels to be ligated, more anatomical parts to be anastomosed and removed, and more wounds to be exposed. A prolonged operation time also increases the possibility of damage to the blood vessels and insufficient hemostasis, thereby significantly elevating the risk of repeated bleeding. In addition, acidic metabolites in the body can accumulate over time, leading to metabolic acidosis and resulting in an increase in oxygen free radicals and mitochondrial damage. This ultimately increases the incidence of secondary hemorrhage. Some surgeons prefer surgery over other options for treatment; however, excessive vascularization could lead to unnecessary complications, such as repeated postoperative bleeding. Liu et al. (4) believe that vascular skeletonization and lymph node dissection could facilitate corrosion of the exposed blood vessels by the digestive fluids and infectious factors exuding from the anastomosis, which can lead to rupture and bleeding of the blood vessels.

In this study, intervention time refers to the time between the detection of bleeding and the initiation of hemostatic treatment. Several factors can lead to a prolonged intervention time. (1) First, most sentinel hemorrhages will first lead to a small amount of bleeding in the abdominal drainage tube or nasogastric tube, and then the bleeding will stop for a short period; these events are often ignored by clinicians. At this time, if relevant examinations and effective hemostasis measures are not carried out, this may lead to massive hemorrhage at timescales up to a few hours or a few days later, with incidence rates of 33.3% to 71.0% (5, 6). Statistics indicate that the mortality rate of patients with sentinel hemorrhage is significantly higher than that of those without sentinel hemorrhage (up to 57%)(1, 7). Therefore, ward clinicians should closely observe the quantity and properties of the drainage fluid in the drainage tube and gastric tube, dynamically perform routine blood tests, analyze coagulation function and arterial blood gas, and monitor real-time changes in the vital signs of their patients. Patients should be under observation for sentinel hemorrhage, and if suspected, effective intervention measures must be immediately taken. (2) Some patients do not show obvious clinical symptoms, and the vital initial signs of postoperative bleeding remain temporarily stable. This might easily be ignored by doctors, thereby prolonging the interval before treatment is initiated. (3) Intraperitoneal hemorrhage in some patients who first undergo non-surgical intervention, such as interventional hemostasis or endoscopy, can be left incompletely cured, leading to extension of the treatment time and an increase in the difficulty of establishing hemostasis. (4) Finally, it cannot be ruled out that the doctor on duty may not take the condition seriously when hemorrhage occurs during their night shift break. Usually, the night shift nurse would report conditions such as a decrease in hemoglobin levels or a change in the vital signs to the resident physicians. However, clinicians may not only fail to understand the serious nature of these conditions, but also fail to report them to their superiors in time. Ultimately, no therapeutic clinical decision is made in such cases, and secondary bleeding continues.

Figure 1 shows the management of postoperative bleeding in this study, which determined the exclusion and inclusion criteria. Surgical hemostasis clearly demonstrated the highest success rate (84.4%) when compared with interventional embolization (26.7%) and endoscopic treatment (50%). For a very long time, secondary surgery has been considered the preferred treatment for postoperative abdominal bleeding. Despite the rise in the popularity of interventional embolization (8–10) and endoscopic therapy (11), the importance of surgery remains undiminished. In this study, 15 patients were administered endovascular treatment (EVT), including 10 patients with visceral arterial hemorrhage. Theoretically, the bleeding site of the superior mesenteric artery and the three major branches of the celiac trunk can be accurately located and embolized to establish “intravascular hemostasis”. However, if the amount of bleeding is large, with a decrease in the blood pressure, vasospasm contraction, blood clot blockage, etc., his may lead to false negative angiography results. If EVT is incomplete, a surgical approach is still the most beneficial option (12). Similarly, electronic endoscopy can identify specific bleeding sites, and isolated bleeding sites can be clamped with vascular clips or electrocoagulation in order to achieve hemostasis (11). However, when hemorrhage is difficult to control, and also in case of a blurred visual field, an informed decision on the second operation should be made (5). Although the surgical approach seems to be difficult and risky due to postoperative adhesions, and is dependent on whether the condition of the patient is critical, it has the advantages of clear vision and complete hemostasis. The key to treatment of active bleeding is early surgical hemostasis. Generally, surgery is the only treatment option for a patient in a critical condition. The principles of surgical treatment are first to stop the bleeding, then to repair the affected part, and finally to perform a third excision of the damaged part. The treatment should be simple, effective, fast, and safe. It is important to note that if hemostasis is not reliably established, bleeding may start again.

According to the 2007 ISGPS consensus (13), the timing of bleeding events can be divided into “early” and “late,” with “early” denoting bleeds occurring less than 24 h postoperatively and “late” denoting those occurring more than 24 h postoperatively. Early hemorrhage is mainly associated with surgical techniques, such as incomplete arterial ligation, vascular clip detachment, anastomotic bleeding, and oozing of blood from abdominal wounds; in contrast, late hemorrhage is mostly attributable to vascular corrosion, usually associated with pancreatic fistula and abdominal infection (1, 14). Previous studies have reported that the mortality rate after secondary surgery in hemodynamically unstable patients with delayed bleeding is as high as 64% (15). Delayed bleeding after abdominal surgery is associated with an increased risk of secondary bleeding and a significant increase in patient mortality following ­secondary bleeding (16, 17).

During abdominal surgery, if multiple organs are removed or additional surgical operations are performed, the patient can experience major trauma and is subjected to a higher risk of vascular and wound hemorrhage. Intra-abdominal infection observed after abdominal surgery is often caused by pancreatic fistula, biliary fistula, intestinal fistula, etc. The underlying mechanisms may involve the activation of digestive enzymes due to local infections and abscesses in the abdominal cavity, which then erode important blood vessels and lead to anastomotic leaks, thereby causing poor patient prognosis (18). When intra-abdominal bleeding fails to drain, it can cause local stasis and blockage to the extent that some bacteria and inflammatory substances will take the opportunity to breed, promoting intra-abdominal infection. Loos (19) et al. found that Pseudomonas aeruginosa and Enterococcus faecium in the peritoneal drainage fluid may have the ability to activate pancreatic enzymes, which can trigger tissue necrosis and vascular erosion, causing abdominal infection (20). This risk can be managed during the operation by removing the accumulated blood completely, eliminating the risk factors for subsequent infection (21). However, this cannot be achieved by conservative treatment or invasive interventional hemostasis measures.

There are certain limitations to the present study. First, as this was a retrospective study, it is susceptible to potential bias and selection bias. Second, retrospective studies like the present one cannot establish causal relationships, but only associations. Despite these limitations, this is the first study to describe and report secondary hemorrhage after abdominal surgery, which has not often been reported on in previous studies. Second, this was a large-scale study with an emphasis on human factors. Third, survival curves were established to illustrate the outcomes of primary bleeding and secondary bleeding, directly reflecting the poor prognosis associated with secondary hemorrhage. Fourth, this study reviewed current practices and outcomes to identify confirmed cases in order to make a valid estimation of multiple relevant risk factors. Fifth, this study examined the timing of hemorrhage and its association with mortality and intervention type. The selection bias in this study was minimized by adjusting all cases using appropriate and rigorous statistical methods.

The analysis of the underlying causes of secondary bleeding in each of the two groups demonstrated the critical role played by subjective human factors. Skilled and careful surgery and strict perioperative treatment and management are the major factors that can help clinicians to largely avoid secondary hemorrhage. Prevention of postoperative hemorrhage requires greater responsibility on the part of surgeons. The occurrence of postoperative hemorrhage in abdominal surgery is not only related to the condition of the patient, but is also closely related to the surgical techniques employed and the proficiency of the chief surgeon (22). During the operation, the surgeon should avoid blind skeletonization of the blood vessels and cover the wound surface with natural omentum or biological agents to the greatest extent possible. In addition, ligatures should be moderately tightened to avoid avulsion of the artery due to the application of excessive force. The placement of the abdominal drainage tube is highly critical in providing key evidence of postoperative hemorrhage. A suitable abdominal drainage tube can not only allow smooth drainage but also provide early warning of intra-abdominal bleeding. In some patients with early bleeding, no significant changes are observed in their vital signs or abdominal signs. Therefore, performing a CT scan even in patients with active bleeding could help the surgeon to localize the bleeding site. Once a hemorrhage occurs, timely identification of the bleeding site and proper selection of an appropriate hemostasis program are the keys to successful treatment. An analysis of specific risk factors for bleeding is important to improve the prognosis of patients with abdominal postoperative hemorrhage.

## Conclusions

Briefly, this study effectively evaluated the risk predictors of secondary hemorrhage and demonstrated that longer operation time and extended intervention time are independent risk predictors. The success rate of surgical hemostasis is higher than those of interventional embolism and endoscopy, indicating that surgical hemostasis is the best option to manage bleeding after abdominal surgery. We conclude that secondary bleeding is positively associated with postoperative mortality. Therefore, efforts by surgeons to improve their operative techniques and ensure proper postoperative management will substantially help in reducing the incidence of postoperative hemorrhage and in gradually preventing morbidity and mortality.

## Data Availability

The original contributions presented in the study are included in the article/Supplementary Material, further inquiries can be directed to the corresponding author/s.
